# Antioxidant activities of saponins extracted from Radix Trichosanthis: an *in vivo* and *in vitro* evaluation

**DOI:** 10.1186/1472-6882-14-86

**Published:** 2014-03-05

**Authors:** Ying Chen, Yonghong Miao, Liyong Huang, Juxiang Li, Haiyan Sun, Yuanzeng Zhao, Jing Yang, Wenke Zhou

**Affiliations:** 1 School of Life Science and Technology, Henan Institute of Science and Technology, Xinxiang, Henan, China; 2 Department of Pediatrics, Henan Provincial People’s Hospital, Zhengzhou, Henan 450003, China; 3 Department of Neurosurgery, the First Affiliated Hospital of Xinxiang Medical University, Weihui, Henan, China

**Keywords:** Antioxidant, Liver, Radix trichosanthis, Radical scavenger, Saponins

## Abstract

**Background:**

Radix Trichosanthis (RT), the dry root tuber of *Trichosanthis kirilowii* Maxim (Cucurbitaceae), is a traditional Chinese medicine. Although a wide range of saponin pharmacological properties has been identified, to our knowledge, this may be the first report to investigate the crude saponins from RT. The purpose of this study was to delineate the antioxidant activity both *in vitro* and *in vivo* by using ethyl acetate (EtOAc), n-butanol, and the mixture of n-butanol and EtOAc fractions.

**Methods:**

*In vitro* antioxidant activity was detected by using DPPH free radical, hydrogen peroxide scavenging, and reducing power assays. After pretreatment with different fractions saponins at 2 mg/kg/d and 3 mg/kg/d of crude drug, respectively, an established CCl_4_ induced acute cytotoxicity model was used to evaluate the *in vivo* antioxidant potential by detection of superoxide dismutase (SOD), malonaldehyde (MDA), lactate dehydrogenase (LDH), and total antioxidant capacity (T-AOC) levels.

**Results:**

The *in vitro* assay showed that the antioxidant activity of all the three fractions was promising. The reducing power of the EtOAc and the mixture of n-butanol and EtOAc extracts increased in a dose dependent manner. However, both the n-butanol and the mixture of n-butanol and EtOAc fractions in low dose exhibited in a time dependent manner with prolonged reaction time. As for hydrogen peroxide scavenging capability, the n-butanol fraction mainly demonstrated a time dependent manner, whereas EtOAc fraction showed a dose dependent manner. However, in case of *in vivo* assay, an increase of SOD and T-AOC and decrease of MDA and LDH levels were only observed in n-butanol (2 mg/kg/d of crude drug) extracts pretreatment group.

**Conclusions:**

RT saponins in n-butanol fraction might be a potential antioxidant candidate, as CCl_4_-induced oxidative stress has been found to be alleviated, which may be associated with the time dependent manner of n-butanol saponins in a low dose. Further studies will be needed to investigate the active individual components in n-butanol extract, *in vivo* antioxidant activities and antioxidant mechanisms.

## Background

A growing body of antioxidant isolated from plant has been identified to date to support health and wellness, as the disbalance of cellular redox homeostasis, which is the reactive oxygen species (ROS) and the antioxidant system, contributes to the pathogenesis of almost all of diseases [1-3]. ROS, composed of superoxide (O_2_¯), hydrogen peroxide (H_2_O_2_), hydroxyl radical (OH¯), and peroxynitrite (ONOO¯), mainly generated by the normal mitochondrial respiration, are critical intracellular signaling messengers. Overload of free radicals may, however, lead to oxidative damage, therefore, it is of great importance for either exogenous or endogenous antioxidants to scavenge the abundant free radicals to protect cellular DNA, proteins and lipid membranes. Even though endogenous antioxidants, such as superoxide dismutase (SOD), catalase (CAT), glutathione peroxidase (GPx), glutathione S-transferase (GSH), and glutathione reductase (GR), are more powerful free radical scavengers than those from diet, however, under pathological conditions, much more of free radical is beyond the capacity of endogenous antioxidants [4]. For example, carbon tetrachloride, being converted to trichloromethyl radical (CCl_3_^**·**^) and trichloromethyl peroxy radical (CCl_3_OO^**·**^) by cytochrome P450 enzyme system in liver, is a well used chemical to induce *in vivo* oxidative stress [5]. Previous reports showed that the formed free radicals may eventually reduce antioxidant enzyme and antioxidant substrates to induce oxidative liver stress [6]. As stated above, it is urgent to explore of antioxidant agents both in food industry and in prevention medicine to reduce the risk of toxicity [7].

Up to now, many herbs have been investigated phytochemically to illuminate their antioxidant actions both in clinical and experimental studies. Radix Trichosanthis (RT), the dry root tuber of *Trichosanthis kirilowii* Maxim (Cucurbitaceae) (voucher number: 15439 in Chinese Field Herbarium) being collected in spring and autumn, is an extensive used traditional Chinese medicine for almost thousand years. The main chemical components of RT are saponins, polysaccharide, amino acid, and protein, but negative for alkaloids and flavonoids [8]. It has been proven that RT protein (trichosanthin, TC) and polysaccharide have many effects, such as antitumor, antivirus, immunomodulatory, as well as abortifacient [9-13]. In addition, the water extracts of RT have been demonstrated to inhibit melanin synthesis by blocking cAMP-induced melanogenesis in B16 cells in a time and dose-dependent manner [14].

As a large family of heterosides compounds of steroid or triterpenoid aglycone (sapogenin), saponins have been shown an increasing of traditional and industrial applications in medicine as anti-inflammatory, molluscicidal, antimicrobial, antispasmodic, antidiabetic, antitumor, antioxidant, as well as adjuvants [15-17], but also in food and cosmetic industry as emulsifiers or sweeteners [18]. A growing body of evidence indicated that some plant saponins have strong antioxidant activities, therefore, they may be the novel potential antioxidant candidates, which may rely on their free radical scavenging abilities [19,20]. Unfortunately, even though some saponins exhibited a strong activity *in vitro*, they may have also been proved less efficient *in vivo*[21]. In view of the knowledge of the efficacy of RT saponins is far more lags of other saponins, such as *Panax Ginseng* and *Glycyrrhiza glabra*[22], with this in mind, we try to improve our understanding of whether RT saponins possesses the *in vitro* and *in vitro* antioxidant activities or not, and the cytotoxicity under proposed dose by Chinese pharmacopoeis.

## Methods

### Chemicals and reagents

DPPH (1,1-diphenyl-2-picrylhydrazyl) was purchased from Sigma- Aldrich Company. Quillaja saponin was purchased from Chendu Must Biotechnology CO., LTD. SOD, malonaldehyde (MDA), lactate dehydrogenase (LDH), and total antioxidant capacity (T-AOC) kit was obtained from Nanjing Institute of Jiancheng Biological Engineering. Mice were purchased from purchased from Xinxiang Medical University. All other agents were analytical of grade.

### Plant materials

RT used in this study was collected from farmland of Henan Institute Science and Technology in autumn, 2012. RT was authenticated by Professor Li Meng from the Department of Botany, Henan Institute Science and Technology.

### Preparation and extract of the crude saponins

Samples was dried at the room temperature and milled into dry power. Ethanol, n-butanol, and ethyl acetate (EtOAc) were used for the extraction. Briefly, 200 g of RT power was extracted three times with 10-fold ethanol. After ethanol solvent being removed by a rotary evaporator, the dried ethanol extract was then dissolved in hot water and partitioned successively with equal volumes of n-butanol and EtOAc respectively. The components of each fraction were subjected to rotary evaporator. All of samples were applied with a silica gel column, eluted with water, 80% ethanol and 100% ethanol in sequence, but only the fraction eluted with 80% ethanol was collected and evaporated. To be more specific, the dry extracts were: n-butanol fraction; EtOAc fraction; and the mixture of n-butanol and EtOAc fraction.

### Determination of total saponins

The total saponins content of different extracts was determined by the vanillin-sulfuric acid method [23]. All of the extracts were mixed with vanillin (8%, w/v) and sulfuric acid (72%, w/v), then incubated at 60°C for 10 min. Being cooled in an ice water bath for 15 min, the absorbance was measured at 538 nm. Quillaja saponin was used as a reference standard and the content of total saponins was expressed as Quillaja saponin equivalents (μg/mg extract) [24].

### DPPH radical scavenging assay

The radical scavenging activity of the plant extracts against DPPH was determined by measuring UV absorbance at 517 nm. The DPPH radical scavenging assay was performed as described by Nazari with slight modifications [25]. In brief, each sample extract (1 ml) at different concentrations (1, 2, 3, 4, and 5 mg/ml) was added to 2 ml DPPH (10 mg/l in methanol). After a 30-min reaction, absorbance was determined. The scavenging ability on DPPH radicals was calculated as follows: Scavenging ability on DPPH radicals (%) = [(A1 - A2)/A1] × 100, where A1 is the absorbance of the control (containing all reagents except the sample extract), and A2 is the absorbance of the sample extract. Vitamin C was used as standard antioxidants.

### Reducing power assay

The reducing power assay was conducted as previously described by Heo [26]. In brief, each sample extract (1 ml) at different concentrations (1, 2, 3, 4, and 5 mg/ml) was first mixed with 0.2 M phosphate buffer (pH 6.6) (2.5 ml), and 1% K_3_Fe(CN)_6_ (w/v) (2.5 ml). After incubation at 50°C for 20 min, trichloroacetic acid (TCA, 10% w/v) (2.5 ml) were added to the mixture followed by centrifugation at 3000 × g for 10 min to stop the reaction. 2.5 ml of the collected upper layer of the mixture was mixed with 2.5 ml distilled water and 0.5 ml ferric chloride (FeCl_3_, 0.1% w/v), and the absorbance of the resulting solution was read at 700 nm against a blank. Vitamin C was used as positive controls.

### Hydroxyl radical scavenging assay

The scavenging ability of the each fraction on hydroxyl radicals was determined according to the method described by Heo with some modifications. Briefly, individual sample extract (1 ml) at different concentrations (1, 2, 3, 4, and 5 mg/ml) was added to the reagent containing 1 ml FeSO4 (1.5 mM), 0.7 ml H_2_O_2_ (6 mM) and 0.3 ml sodium salicylate (20 mM) [26]. After incubation for 1 h at 37°C, absorbance of the reaction mixture was read at 562 nm. The scavenging ability of hydroxyl radicals was calculated using the following equation: Scavenging ability on hydroxyl radicals (%) = [(A1 - A2)/A1] × 100, where A1 is the absorbance of the control reaction (containing all reagents except the sample extract), and A2 is the absorbance of the sample extract. Again, vitamin C was used as positive controls.

### Animals and treatment

The animal use and care protocols were approved by Institutional Animal Care and Use Committee (IACUC) of Xinxiang Medical University. Forty-eight adult male Kunming mice weighing from 18 to 20 g were purchased from Xinxiang Medical University. All animals were required to undergo institutional quarantine for 7 days prior to use. The environment for animal housing was equipped with controlled temperature (22 ± 3°C), humidity (40% – 70%), and a 12 h light/dark alternation. They were given standard pellet diet and water *ad libitum*. To study the antioxidant effects of saponins, mice were equally divided into eight groups (*n* = 6). Group I (control group) received saline (0.9%) intragastrically (2 ml/kg body weight) during the experiment. Group II (an oxidant control group) was given saline (0.9%) intragastrically (2 ml/kg body weight) for 15 days before CCl_4_ intoxication. Group III and IV (EtOAc fraction treatment group with a dose of 2 mg/kg/d and 3 mg/kg/d of crude drug, respectively), Group V and VI (n-butanol fraction treatment group with a dose of 2 mg/kg/d and 3 mg/kg/d of crude drug, respectively), and Group VII and VIII (the mixture of n-butanol and EtOAc fraction treatment group with a dose of 2 mg/kg/d and 3 mg/kg/d of crude drug, respectively) were administered intragastrically for 15 days before CCl_4_ injection. The mice received an intraperitoneal injection of CCl_4_ (0.2 mL/mouse of 0.1% CCl_4_ solution in olive oil) 13 h before the final administration in different groups, except the normal control groups (group I), which was intraperitoneally treated with an equal amount of olive oil.

### Tissue collection

After 24 h of CCl_4_ and olive injection, all the animals were sacrificed. For determination of oxidative stress, the fresh liver tissue was collected and homogenized in 100 mmol KH_2_PO_4_ buffer containing 1 mmol EDTA (pH 7.4). After centrifugation at 12,000 × g for 30 min at 4°C, the supernatant was collected and stored at −70°C for further studies. Protein concentration was determined by Bradford method.

### LDH assay

The supernatant of all the samples was collected after homogenate and the LDH content was determined using an LDH assay kit according to the manufacturer’s instructions. LDH cytotoxicity was calculated using OD as LDH cytotoxicity (U/g protein) = (OD sample - OD blank)/(OD standard solution – OD blank standard solution) × standard solution concentration/sample protein concentration.

### MDA assay

MDA level was measured by the thiobarbituric acid method with MDA assay kit according to the manufacturer’s instructions. Samples (10 μl) were suspended in 200 μl thiobarbituric acid (TBA) reagent and heated at 95°C for 40 min. After cooling, the reaction mixture was centrifuged at 6,000 × g for 10 min, and TBARS equivalent in supernatants monitored at 532 nm. MDA was calculated using OD as MDA level (nmol/mg protein) = (OD sample − OD blank)/(OD standard solution – OD blank standard solution) × standard solution concentration/sample protein concentration.

### SOD assay

The SOD activity of liver tissue was estimated using SOD assay kit according to the manufacturer’s instructions. Briefly, samples (30 μl) were mixed with 3.3 ml of reaction mixture containing xanthine oxidase to oxidizing of NBT by O_2_^•−^ monitored at 550 nm. SOD was calculated using OD as SOD level (U/mg protein) = (OD blank − OD sample)/OD blank/50% × dilution of reaction system/sample protein concentration. One unit of SOD activity was defined as that producing 50% dismutation of O_2_^•−^ radical.

### T-AOC assay

Ferric-reducing antioxidant power (FRAP) was measured by using total antioxidative capacity (T-AOC) commercial kit according to the manufacturer’s instructions. The stable color of the Fe^2+^ -o-phenanthroline complex was measured at 520 nm. T-AOC was expressed in U/mg protein where 1 U is defined as an increase in absorbance (A520) of 0.01/min/mg protein at 37°C. T-AOC was calculated using OD as T-AOC level (U/mg protein) = (OD sample − OD blank)/0.01/30 × dilution of reaction system/sample protein concentration.

### Statistical analysis

All of the statistical analysis was performed using the Statistical Package for the Social Sciences (SPSS Inc., Chicago, IL) program. All data were reported as means ± SD of three independent experiments and were analyzed by oneway ANOVA followed by LSD multiple comparison post hoc analysis. For all comparisons, *P* < 0.05 was considered statistically significant.

## Results and discussion

### Total saponin content

Being widely distributed amongst plants, saponins have long been regarded as phytochemical material to protect plant against pathogens. Therefore, it is no doubt that saponins function as potential medicinal candidates. In this study, total saponins content is reported as Quillaja saponin equivalent to standard curve (y = 1.847x + 0.0884, R^2^ = 0.9905). The total saponins of RT from n-butanol, EtOAc, and the mixture of n-butanol and EtOAc extracts were 1.824, 1.678, and 2.3998 mg, respectively.

### DPPH radical scavenging activity

**Figure 1:**
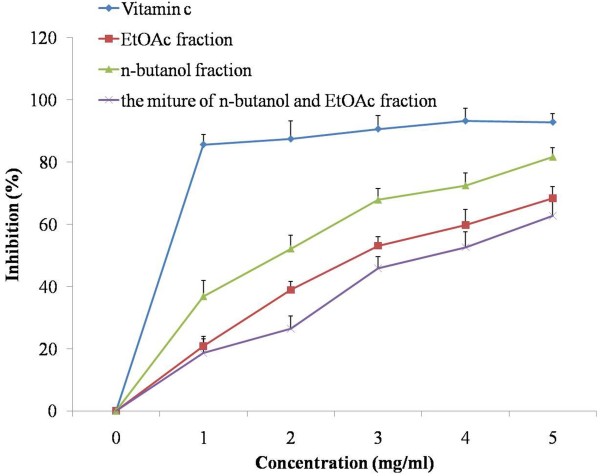
**DPPH radical scavenging activities of the extracts from radix trichosanthis and the positive controls.** Data were the mean of three independent experiments.

DPPH is a relatively stable free radical. DPPH radical scavenging activity is a widely used method to evaluate the free radical scavenging ability of various samples. This method is based on the reduction of DPPH in the presence of a radical scavenger or hydrogen donors due to the formation of non-radical form of DPPH-H. As shown in Figure 1, all three extracts scavenged DPPH radicals in a dose-dependent manner. The IC_50_ for DPPH inhibition was 0.9908 ± 0.08, 2.9246 ± 0.15, 2.8428 ± 0.11, and 4.3972 ± 0.23 mg for vitamin C, EtOAc, n-butanol, and the mixture of n-butanol and EtOAc fractions, respectively. Inhibition was 4 mg/ml for vitamin C, 5 mg/ml for extracts EtOAc, n-butanol, and the mixture of n-butanol and EtOAc, respectively. Among all the three fractions, n-butanol extract has lowest IC_50_ compared with the others, suggesting that this fraction may be the potential antioxidant reagent.

### Reducing power

**Figure 2:**
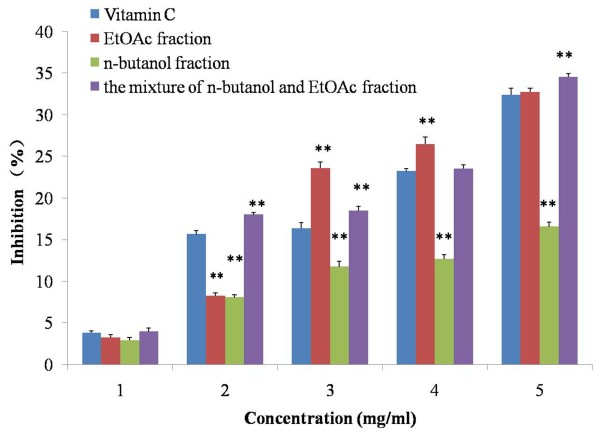
**Reducing power of the extracts from radix trichosanthis and the positive controls.** Data were the mean of three independent experiments. ^**^P < 0.01 versus VC.

Figure 2 shows the reducing power potentials of the three extracts of the test plant in comparison with a standard vitamin C. In the reducing power assay, the presence of antioxidants in the sample would result in the reduction of Fe^3+^ to Fe^2+^ by donating an electron. The amount of Fe^2+^ complex can then be monitored by measuring the formation of Perl’s blue at 700 nm. Increasing absorbance indicates an increase in reductive ability. The results showed that the reducing power was increased with the concentration increases (Figure 2). However, contrary to the dramatically increased reducing power in EtOAc and the mixture of n-butanol and EtOAc fractions, the reducing power of n-butanol fraction was lower than that of control group until the end of the experiment. No difference was observed between vitamin C group and the three RT saponin extracts at 1 mg/ml concentration (*P* > 0.05) when analysed statistically. With the increased concentration, there were significant difference between all three extracts at 2 mg/ml concentration (*P* < 0.05 *vs*. control), but the levels of n-butanol and EtOAc fractions were lower than that of control group, whereas the reducing power in the mixture fractions was higher above the control. Once the concentration increased from 3 to 5 mg/ml, high levels reducing power and significant difference was observed in EtOAc and the mixture extracts (*P* < 0.01). The results indicated that EtOAc and the mixture of n-butanol and EtOAc extracts, but not n-butanol fraction, showed robust reducing power in a dose dependent manner.

**Figure 3:**
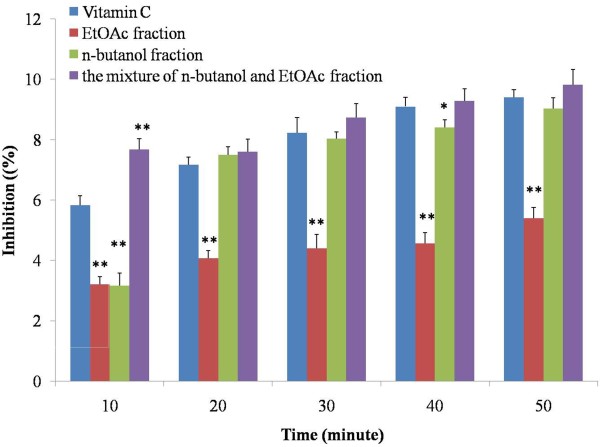
**Reducing power of the extracts from radix trichosanthis and the positive controls at 1 mg/ml with different reaction time.** Data were the mean of three independent experiments. ^*^P < 0.05 versus VC; ^**^P < 0.01 versus VC.

More interestingly, when the reaction time was controlled at 10 min, the three extract groups showed significant difference with control group (*P* < 0.01) (Figure 3). The reducing power of extracts of EtOAc and n-butanol was lower than that of control group, whereas the level of the mixture of n-butanol and EtOAc fraction was higher than that of control. However, with the prolonged reaction time, the reducing power was gradually increased both in n-butanol, and the mixture of n-butanol and EtOAc groups reaching to the control level (*P* > 0.05), except that of EtOAc fraction (*P* < 0.01). Among the two increased group, namely n-butanol and the mixture of n-butanol and EtOAc extracts, the reducing power of n-butanol extract was dramatically increased after 20 min until to 50 min, even though they all showed no significant difference with control group. As for the mixture of n-butanol and EtOAc extract, the increasing reducing power maintained a stable level. These results suggested that at a low concentration, both the n-butanol and the mixture of n-butanol and EtOAc fractions showed a strong reducing power in a time dependent manner.

### Hydrogen peroxide scavenging

**Figure 4:**
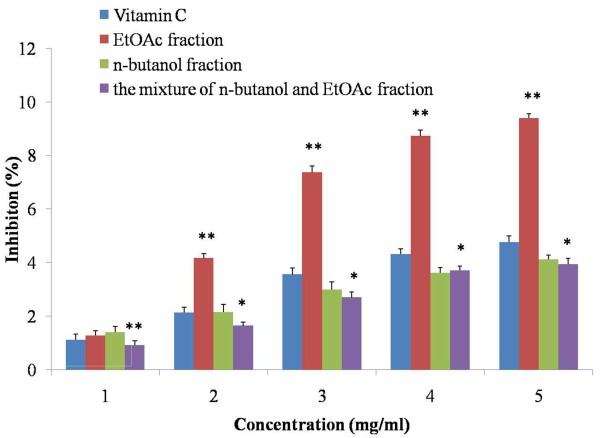
**Hydrogen peroxide scavenging activity of the extracts from radix trichosanthis and the positive controls.** Data were the mean of three independent experiments. ^*^P < 0.05 versus VC; ^**^P < 0.01 versus VC.

Hydrogen peroxide is an important reactive oxygen species due to its ability to penetrate biological membrane. Therefore, hydroxyl radical is regarded as one of the most reactive free radicals, which can induce severe damage of biomolecules [27]. The ability of the extracts to effectively scavenge H_2_O_2_ was determined according to the method of Ruch as vitamin C as standards. The trend of each extract being capable of scavenging hydrogen peroxide was different with that of reducing power (Figure 4). At 1 mg/ml, only n-butanol extract showed the higher capacity of scavenging hydrogen peroxide than that of control group with significant difference (*P* < 0.05). However, with the concentration increased from 2 to 5 mg/ml, the hydrogen peroxide scavenging ability in n-butanol extract was reduced, just as that of the mixture fraction (*P* < 0.05 *vs*. control). Conversely, EtOAc group was increased dramatically, and there was significant difference (*P* < 0.01), indicating that EtOAc fraction exhibited a strong scavenging hydrogen peroxide capability in a dose dependent manner.

**Figure 5:**
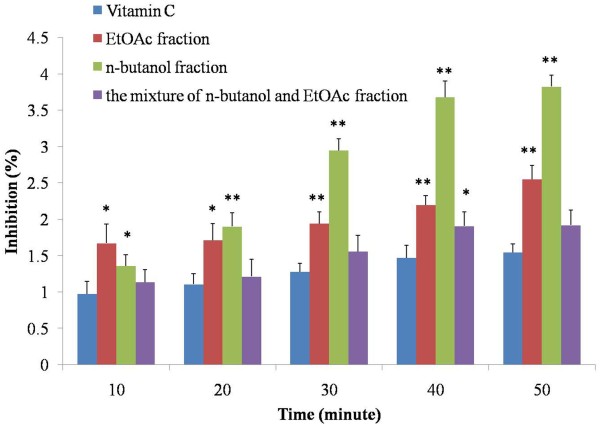
**Hydrogen peroxide scavenging activity of the extracts from radix trichosanthis and the positive controls at 1 mg/ml with different reaction time.** Data were the mean of three independent experiments. ^*^P < 0.05 versus VC; ^**^P < 0.01 versus VC.

Once we prolonged the reaction time, the scavenging ability of all three groups was increased and higher than that of control, particularly of EtOAc and n-butanol fractions (Figure 5). At 10 min, the EtOAc and n-butanol fractions showed significant difference (*P* < 0.05). With the time expanded, the scavenging ability of n-butanol extract was increased dramatically at 20 min (*P* < 0.01) than that of in EtOAc group (*P* < 0.05). After that time, the significant difference was observed (*P* < 0.01) in both EtOAc and n-butanol fractions. Even though the mixture of n-butanol and EtOAc extract showed no significant difference, its scavenging ability was also higher than that of control group, indicating that the scavenging ability of all three fractions was in a time dependent manner. Taken together, the n-butanol fraction mainly demonstrated a time dependent manner, whereas EtOAc fraction showed a dose dependent manner.

### The cytotoxic effect of RT saponins

Up to now, there are few reports on saponins cytotoxicity on normal cells [28,29]. Therefore, it is urgent to clarify whether RT saponins have the cytotoxicity or not *in vivo*, even though RT saponins have been proved to be a strong antioxidant *in vitro*. LDH activity is widely used as a cytotoxic marker. Using this assay, we detected a cytotoxic effect of RT saponins in CCl_4_ induced mouse model. As shown in Table 1, LDH activity was very low in the control group. After CCl_4_ treatment, LDH levels were significantly increased (*P* < 0.05). However, a significant decrease in LDH activity was only observed in pretreatment of RT saponins group V (*P* > 0.05 *vs*. group I; *P* < 0.05 *vs*. group II), showing that group V has the most hepato-protective potential and less cytotoxicity. Unfortunately, the others exhibited higher LDH activity than that of CCl_4_ treated group (*P* > 0.05 *vs*. group II), indicating that EtOAc (group III and IV), n-butanol (high dose) (group VI), and the mixture of n-butanol and EtOAc fraction (group VII and VIII) might also aggravate the liver injury.

**Table 1:** Effect of saponins on assessment of oxidative stress

**Group**	**LDH (U/g)**	**SOD (U/mg protein)**	**T-AOC(U/mg protein)**	**MDA(nmol/ mg protein)**
Group I	295.9 ± 45.3	87.6 ± 12.3	1.8 ± 0.20	1.3 ± 0.21
Group II	753.1 ± 53.7^**^	15.6 ± 3.6^**^	0.7 ± 0.12^**^	7.1 ± 0.28^**^
Group III	2489.3 ± 104.8^**##^	93.8 ± 10.5^##^	2.1 ± 0.22^*##^	2.4 ± 0.18^**##^
Group IV	2156.8 ± 95.3^**##^	113.2 ± 14.2^**##^	1.7 ± 0.10^##^	3.6 ± 0.24^**##^
Group V	315.2 ± 23.5^##^	547.4 ± 20.5^**##^	8.0 ± 0.25^**##^	0.2 ± 0.05^**##^
Group VI	1191.7 ± 51.7^**##^	103.8 ± 11.3^*##^	1.1 ± 0.21^**##^	1.1 ± 0.10^##^
Group VII	1479.0 ± 62.4^**##^	92.9 ± 9.8^##^	1.9 ± 0.16^##^	1.2 ± 0.13^##^
Group VIII	4309.3 ± 112.4^**##^	10.6 ± 2.7^**#^	0.6 ± 0.09^**^	10.5 ± 0.54^**##^

Data are mean ± SD (*n* = 6)

^*^P < 0.05 *vs*. group I ; ^**^P < 0.01 *vs*. group I; ^##^*P* < 0.05 *vs*. group II.

### Assessment of oxidative stress of RT saponins

It has been shown that the CCl_4_ induced liver injury was based on its inhibition ability of anti-oxidant enzyme. Therefore, in this study, MDA, SOD, and T-AOC were used as oxidative stress markers to evaluate the antioxidant activity of RT saponins (Table 1). The results showed that MDA increased and SOD and T-AOC decreased in CCl_4_ induced liver injury group (group II) (*P* < 0.05 *vs*. group I). After pretreatment with RT saponins, decreased MDA and increased SOD and T-AOC activity was observed (group III to VII) (*P* < 0.05 *vs*. group II), but not in group VIII, in which MDA increased and SOD and T-AOC decreased even than that of CCl_4_ treated model (*P* < 0.05). Unexpected, in group V, MDA decreased and SOD and T-AOC increased even higher than that of control group (*P* < 0.05), indicating that the saponins in group V are more effective in alleviating oxidative stress in mouse with liver injury.

## Conclusions

Although RT has long been regarded as a traditional medicinal herb in China, to our knowledge, this is the first report to investigate the crude saponins content, antioxidant effect, as well as cytotoxic effect of RT. As for reducing power assay, EtOAc and the mixture of n-butanol and EtOAc extracts showed robust reducing power in a dose dependent manner, whereas n-butanol fraction exhibited in a time dependent manner. From the point of hydrogen peroxide scavenging capability, EtOAc fraction exhibited in a dose dependent manner, contrary to n-butanol fraction’s time dependent manner.

On the other hand, some researchers suggested that an intake of antioxidants, which inhibits or delays the oxidation of molecules *in vitro*, might really work *in vivo*. In this report, n-butanol fraction with low dose showed the most effective activating endogenous antioxidant capability, whereas the mixture of n-butanol and EtOAc treatment group exhibited less antioxidant activity *in vivo*. Taken together, n-butanol fraction might be a potential antioxidant candidate, as the lower dose the less cytotoxicology. This hypothesis was proved by the *in vivo* LDH test.

At present study, it was clearly established that n-butanol fraction of RT has the antioxidant potency both *in vitro* and *in vivo*. Further studies will be needed to investigate the active individual components in n-butanol extract, *in vivo* antioxidant activities and antioxidant mechanisms.

## Competing interests

The authors declare that they have no competing interests.

## Authors’ contributions

HS and JY prepared the extracts and carried out the total saponins content. YM and YZ detented the *in vitro* antioxidant studies. YC worked on the cytotoxicity assay as well as *in vivo* antioxidant assays. Liyong Huang established the mouse model. YC, LH, and WZ analyzed the data. YM, YC and JL evaluated the data and edited the manuscript. All authors have read and approved the final manuscript.

## Pre-publication history

The pre-publication history for this paper can be accessed here:

http://www.biomedcentral.com/1472-6882/14/86/prepub
